# Gene manipulation in *Oenococcus oeni* based on a newly applicable gene gun technology

**DOI:** 10.3389/fmicb.2025.1545266

**Published:** 2025-02-17

**Authors:** Mengrong Chen, Qiling Chen

**Affiliations:** College of Food Science and Pharmacy, Xinjiang Agricultural University, Urumqi, Xinjiang, China

**Keywords:** *Oenococcus oeni*, gene gun, detonation nanodiamonds, stable genetic transfer, wine

## Abstract

*Oenococcus oeni* is an important engineering microbe in winemaking. Detailed knowledge of its growth and metabolism in harsh wine environments could contribute to breeding elite *O. oeni* varieties. However, further studies on this topic do not appear to be sustained due to the lack of stable and reproducible technology to perform gene manipulation on *O. oeni*. Therefore, this research was designed to study gene function by exploring a newly applicable transformation technique that could perform stably and reproducibly on *O. oeni*. By using gene gun technology with detonation nanodiamonds as a plasmid DNA carrier, we achieved stable and reproducible plasmid DNA transformation in *O. oeni*. In addition, the plasmid with the chloramphenicol resistance gene allowed *O. oeni* SX-1b to thrive in chloramphenicol medium.

## Introduction

*Oenococcus oeni* is an important engineering microbe in winemaking due to its excellent fermentative behavior (Bech-Terkilsen et al., 2020; Cappello et al., 2014; Guzzon et al., 2009; Liu et al., 2024). Selected strains of this species have been traditionally chosen as microorganisms for use in wine starter cultures (Grandvalet, 2017; St'Ana and Lemos, 2024; Zhang et al., 2024). In-depth investigations of the functional genes involved in growth and metabolism in harsh wine environments of this species could contribute to breeding more elite *O. oeni* varieties. However, despite multiple techniques available for the transformation of bacteria, several species, including *O. oeni*, are still resistant to the introduction of foreign DNA (Beltramo et al., 2004). However, the plasmid pSR7Rep, which was constructed using the pRS7 obtained from *O. oeni* itself, could not achieve the transformation (Rodriguez et al., 2015). Therefore, current research is primarily focused on “big data omics” that encompass genomics, metabolomics, transcriptomics and proteomics (Chen et al., 2022; Liang et al., 2020; Liu et al., 2017; Margalef-Catala et al., 2016; Sternes et al., 2017) or gene function validation by heterologous expression (Morel et al., 2001; Qi et al., 2020; Yuan et al., 2019; Zhao et al., 2019a,b; Zheng et al., 2024). Thus, stable and reproducible transformation of foreign DNA is a prerequisite to achieving the genetic manipulation of *O. oeni*.

Gene gun technology was developed to enhance DNA delivery, and gene gun-mediated gene transfer is widely used in plants, yeast, gene vaccines and gene therapy (Ghogare et al., 2021; Imai, 2021; Jafari et al., 2012; Slon-Campos et al., 2020). Moreover, *O. oeni* has a diameter of <500 nm (Wang et al., 2019), and nanoscale material is needed as a vehicle (Osipov et al., 2020; Stehlik et al., 2016). Therefore, this research was designed to tentatively study the gene functions of *O. oeni* based on the precondition that gene gun combined with detonation nanodiamonds (DNDs) could achieve a stable and reproducible transformation into *O. oeni*.

## Results and discussion

### Gene gun combined with DNDs achieved stable and reproducible transformation of exogenous plasmids in *O. oeni*

*O. oeni* is an important engineering bacterium in wine making (Bech-Terkilsen et al., 2020; Guzzon et al., 2009). Thus, intensive study on this useful industrial microorganism is necessary. However, in-depth research is sluggish and proceeds gradually due to the lack of efficient gene-transfer techniques (Beltramo et al., 2004; Rodriguez et al., 2015). Therefore, stable and reproducible transformation of foreign DNA is an important prerequisite to enable genetic manipulation of *O. oeni*.

**Figure 1:**
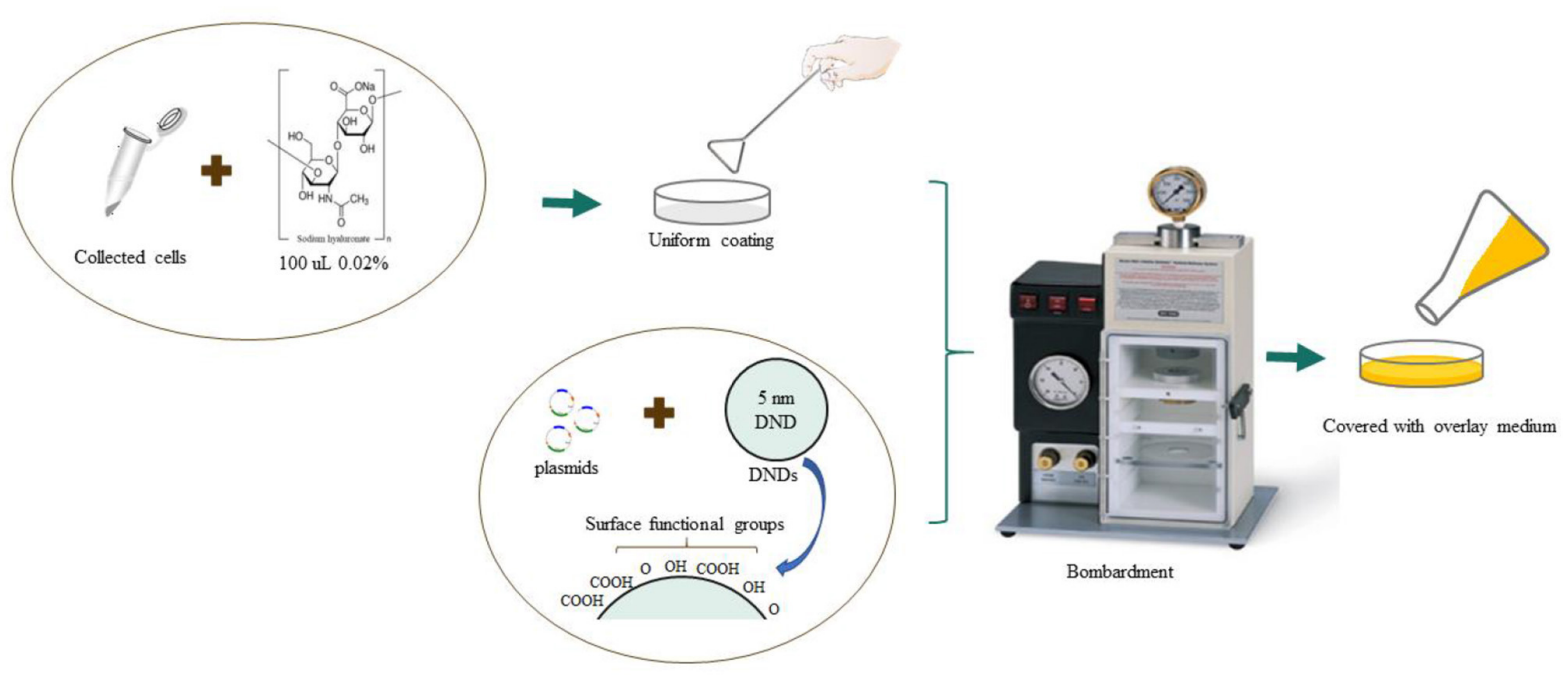
Technical processes involved in transforming detonation nanodiamond (DND)-coated plasmids into *Oenococcus oeni* by gene gun.

Figure 1 provides a flowchart of the particle bombardment on *O. oeni* by the gene gun. The detailed procedure is presented in the materials and methods section. To ensure the successful transformation of the exogenous plasmid coated with DNDs by gene gun on *O. oeni*, a recombination plasmid pLCNICK-*citP* with a size of 14 kbp and carrying an exogenous gene that is specific for *Lactiplantibacillus plantarum* WCFS1 and therefore must be absent in *O. oeni*, was transformed into *O. oeni* SX-1b, SX-1a and SD-2a cells collected at various growth stages. After bombardment, the cells were transferred to 2.8% agar ATB with erythromycin resistance and incubated in at 26°C.

**Figure 2:**
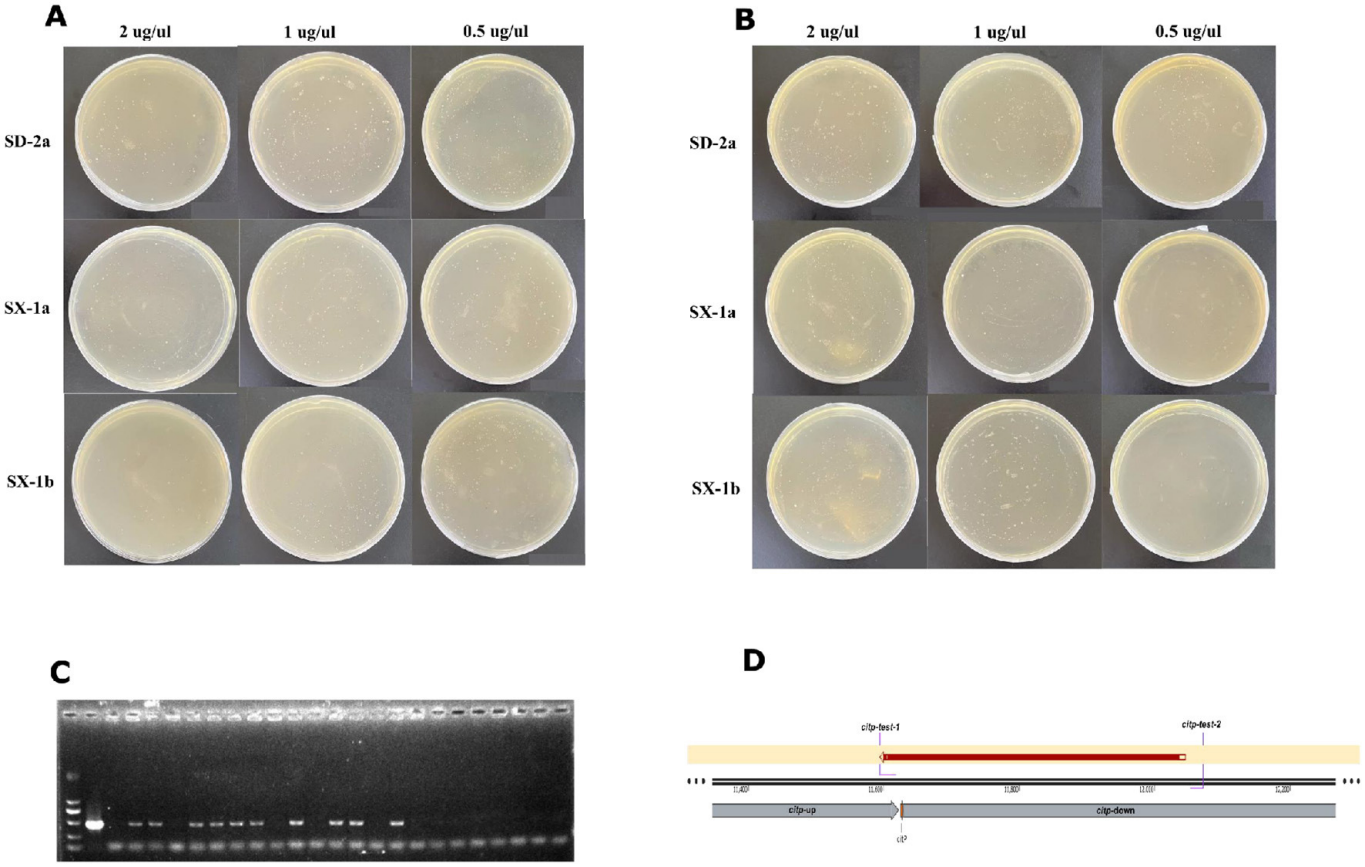
Results of the bombardment experiment of *O. oeni* by gene gun. Transformed colonies appeared on the selection plates after 7 days of incubation. **(A)** Transformed cells collected at 20 h; and **(B)** transformed cells collected at 45 h. **(C)** Randomly selected transformants were verified by colony PCR, and the 477 bp products were checked via electrophoresis to confirm the correct construct. **(D)** Single colony clones were validated by DNA sequencing, with the sequence perfectly matching the criteria.

Transformed colonies appeared after 7 days of incubation. As shown in Figures 2A,B, all plates grew dense colonies (>400), regardless of the different *O. oeni* strains (SX-1a, SX-1b and SD-2a), gradient concentrations of plasmid (2 μg/μl, 1 μg/μl and 0.5 μg/μl), or different collection times (20 and 45 h). Except for plate bombardment with 0.5 μg/μl plasmid, SX-1b cells collected at 45 h grew to fewer than 10 single colonies. Then, single colonies were randomly selected and identified through Sanger sequencing with pairs of primers (*citP*-test) (Figures 2C,D). Overall, the transformation efficiency was approximately 40% by colony PCR at either low or high concentrations of plasmid. Thus, gene gun combined with DND technology may be employed as an efficient transformation method for *O. oeni*.

### Overexpression of the chloramphenicol-gene improves survival of *O. oeni*

**Figure 3:**
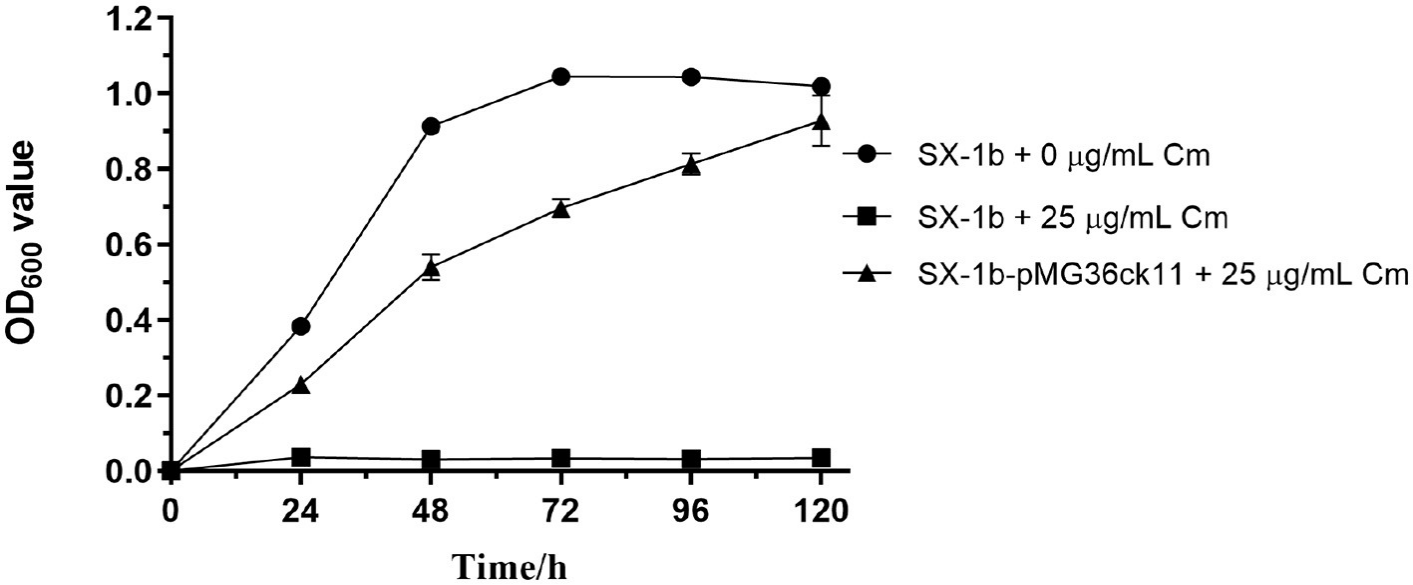
Proliferative capacities of *O. oeni* mutants and wild-type strains cultivated in chloramphenicol medium. The absorbance values (OD_600_ nm) were calculated as the tested samples' OD reading minus the negative control's OD reading. Error bars represent the SD of three biological repeats.

The plasmid pMG36ck11 (modified from plasmid pMG36e, and have a chloramphenicol resistance gene) was successfully introduced into *O. oeni* SX-1b, with colonies emerging after over 12 days on chloramphenicol selection plates. This delay in colony formation is likely due to the inhibitory effects of chloramphenicol on protein synthesis, which can slow down bacterial growth. Growth curve analyses (Figure 3) further supported these findings, showing that the mutant strain *O. oeni* SX-1b-pMG36ck11 was able to thrive in chloramphenicol, unlike the wild strain. This difference in growth behavior between the wild and transformed strains provides strong evidence of successful plasmid transfer and expression of the chloramphenicol resistance gene.

### Quantitative data did not fit the phenotype

Contrary to expectations, the qPCR results were disappointing and discouraging because the Ct values were very high for the antibiotic resistance gene [mean Ct value was 34.67 for the chloramphenicol gene, whereas the mean Ct value was 17.09 for the reference gene ddl (Costantini et al., 2011)], although significant differences were found for the survival ratio between mutants and wild *O. oeni* SX-1b (Figure 3). This also indicates from another perspective that there are mechanisms within *O. oeni* cells that can recognize and rapidly degrade foreign genes. Such mechanisms may protect the cells from potential toxic effects by limiting the expression of foreign genes (Leinyuy et al., 2023).

### CRISPR-mediated gene knockout failed in *O. oeni*

The knockout plasmid pLCNICK-1070 was created for *O. oeni* SX-1b and successfully introduced into the bacteria. Transformants were cultured in ATB medium with erythromycin, but gene knockout was not achieved. This may also due to the strain's weak acceptance of foreign DNA.

### Challenges persist in gene manipulation in *O. oeni*

In summary, the results appear to indicate that the lower expression of genes located on the plasmid and transferred into *O. oeni* is not due to the vector itself or the source of the DNA. In addition, previous research in our laboratory showed that *O. oeni* transferred with a GFP fluorescence plasmid did not present GFP fluorescence. It seems that *O. oeni* may have a restriction system that could interfere with exogenous genes, as speculated by previous researchers (Beltramo et al., 2004; Rodriguez et al., 2015), and this possibility deserves further investigation. In addition, we speculated that although this restriction system might strongly inhibit exogenous DNA replication, exogenous DNAs could continue to be functional, which was also supported by the findings reported by RNA silencing (Darsonval et al., 2016).

### Conclusion

Gene gun technology with detonation nanodiamonds successfully and consistently transformed DNA into *O. oeni* cells. Overexpressing the pMG36ck11 plasmid (which contains a chloramphenicol resistance gene) enabled *O. oeni* SX-1b to survive in chloramphenicol medium. The reduced expression of plasmid genes may be linked to a restriction system in *O. oeni*, warranting further study. This research establishes a basis for gene manipulation in *O. oeni* species.

## Materials and methods

### Transformation of exogenous genes by gene gun

*O. oeni* was grown in ATB medium (pH 4.8, 26°C) and subcultured twice before being bombarded by a gene gun. One milliliter of cells in logarithmic phase (20 h for mid-logarithmic phase and 45 h for late logarithmic phase, with the OD_600_ adjusted at 1.0 by dilution or concentration with fresh ATB medium) was harvested by centrifugation (8000 rpm, 10 min) and washed with distilled water. Subsequently, the cells were collected by centrifugation again and then suspended in 0.02% sodium hyaluronate solution. Plasmids for the bombardment experiment were concentrated in plasmid DNA (2, 1, and 0.5 μg/mL) by freeze-drying. The collected cells were transiently transformed by particle bombardment using a gene gun (Bio–Rad, Biolistic PDS-1000/He). The experimental protocol for bombardment is based on the work of Grichko et al. (2006), with minor modifications.

The DNDs were purchased from Sigma-Aldrich (America, Cat No. 900180, 5 nm average part. size (DLS), 10 mg/mL in H_2_O, carboxylated). To prepare DND carriers for plasmid DNA delivery by particle bombardment, 0.1 mL DND hydrosol was diluted by adding 0.9 mL of distilled water. Then, the DNDs were repeatedly washed in distilled water through cycles of centrifugation and sterilized by autoclaving to generate 1 mL of approximately 10% hydrosol, which was stored at 4°C until use.

On the day of the experiment, 5 μL of different plasmid concentrations in Tris buffer (pH 8), 50 μL of 2.5 M CaCl_2_, and 20 μL of freshly prepared 0.1 M spermidine were added to 50 μL of DND hydrosol while continuously vortexing. The suspension was vortexed at low speed for 5 min and kept on ice for 10 min, and plasmid-coated DNDs were collected by centrifugation, washed in absolute ethanol, and resuspended in 50 μL of absolute ethanol.

Cells were bombarded with a Biolistic PDS-1000/He gene gun system (Bio–Rad) according to the manufacturer's instructions. The suspensions were settled in a sample disc and then placed into the target chamber of the gene gun chamber. Ten microliters of the suspension was placed on the center of a Bio–Rad macrocarrier disk and air-dried. A Bio–Rad PDS-1000/He instrument was operated according to the manufacturer's instructions using 1100 psi He pressure and approximately 28 in. Hg vacuum. Penetration of DNDs along with plasmids into *O. oeni* was confirmed by PCR with gene-specific primers. When the vacuum in the target chamber was below 28 MPa, the strains in the sample tray were bombarded with DND particles (5 nm average size) with plasmid. Then, the cooling (approximately 40°C) solid medium was poured onto the plastic dished with the bombarded strains. The plates were then incubated at 26°C.

### Plasmid construction

The *O. oeni* strains used in this research were collected and preserved in our laboratory. Plasmids used in this research: pLCNICK (encodes erythromycin resistance and kanamycin double resistance) and pMG36ck11 (modified from plasmid pMG36e by adding a kanamycin resistance gene, replacing the P32 promoter with P11, and replacing the erythromycin gene with the chloramphenicol gene). The primers used in this study are shown in Table 1.

**Table 1:** Primers used in this study.

	**Primer name**	**Primer sequence**
Primers for plasmid pMG36ck11 construction	cat_1	TCAGCACAGTTCATTATCAACCgaggcatatcaaatgaactt
	cat_2	GCAAACCCGTATTCCACGATTAttataaaagccagtcattagg
	pMG36ek11-cat-1	ggttgataatgaactgtgctga
	pMG36ek11-cat-2	taatcgtggaatacgggtttgc
Colony PCR	citP-test-1	TGATGAGTAAGTATGAGGAGGAA
	citP-test-2	TGACCGAATGGACATGCTAT
Quantitative Real-Time PCR	Ddl-1	GGTTCTTCGGTTGGCGTTTCTC
	Ddl-2	CTGCCCAGGAGCCCAATGTG
	Cat-1	gcattttcaggtataggtgt
	Cat-2	attctctggtatttggactc

PrimerSTAR^®^ Max DNA Polymerase (Takara) was used to amplify clones of genes from *O. oeni* fragments, which were ligated into vectors by a NovoRec^®^ Plus PCR one-step cloning kit (NR005, Novoprotein). The application and ligation system and program followed the manufacturer's instructions. DNA was recovered using a DNA purification kit (Tiangen Biotech, Beijing, China) for purification of PCR DNA fragments. The plasmids were extracted using a Plasmid Extraction kit (Tiangen Biotech, Beijing, China).

### Survival rate test

Cells were grown to an OD_600_ of 1.0 prior to induction, then the cells were harvested by centrifugation and resuspended in distilled water twice. Mutants and wild strain were cultured in the pH 4.8 ATB medium contained 25 μg/mL chloramphenicol with a 4% inoculation amount, besides wild SX-1b was cultured in pH 4.8 ATB medium without any resistance.

### Quantitative real-time PCR

Culture samples (5 mL) were collected at the mid-logarithmic phase of growth after introduction to ATB medium (containing 25 μg/mL chloramphenicol). RNA was extracted using AG RNAex Pro Reagent, and total RNA was reverse transcribed using an Evo M-MLV RT Kit with gDNA Clean for qPCR II. Fluorescence quantitative PCR and fluorescence quantitative PCR were performed according to the procedure of the SYBR^®^ Green Premix Pro Taq HS qPCR Kit. All kits were purchased from Accurate Biotechnology (Hunan) Co., Ltd.

## Data Availability

The original contributions presented in the study are included in the article/supplementary material, further inquiries can be directed to the corresponding author.
